# Challenges and advances in biotechnological approaches for the synthesis of canolol and other vinylphenols from biobased *p*-hydroxycinnamic acids: a review

**DOI:** 10.1186/s13068-023-02425-w

**Published:** 2023-11-14

**Authors:** Anne Lomascolo, Elise Odinot, Pierre Villeneuve, Jérôme Lecomte

**Affiliations:** 1 Aix Marseille Univ., INRAE, UMR1163 BBF Biodiversité et Biotechnologie Fongiques, 13009 Marseille, France; 2 OléoInnov, 19 rue du Musée, 13001 Marseille, France; 3 CIRAD, UMR Qualisud, 34398 Montpellier, France; 4 Qualisud, Univ Montpellier, Avignon Université, CIRAD, Institut Agro, IRD, Université de La Réunion, Montpellier, France

**Keywords:** Biotechnological process, Canolol, Hydroxycinnamic acid, Phenolic acid decarboxylase, Rapeseed meal, Sinapic acid, Vinylphenol

## Abstract

*p-*Hydroxycinnamic acids, such as sinapic, ferulic, *p*-coumaric and caffeic acids, are among the most abundant phenolic compounds found in plant biomass and agro-industrial by-products (e.g. cereal brans, sugar-beet and coffee pulps, oilseed meals). These *p-*hydroxycinnamic acids, and their resulting decarboxylation products named vinylphenols (canolol, 4-vinylguaiacol, 4-vinylphenol, 4-vinylcatechol), are bioactive molecules with many properties including antioxidant, anti-inflammatory and antimicrobial activities, and potential applications in food, cosmetic or pharmaceutical industries. They were also shown to be suitable precursors of new sustainable polymers and biobased substitutes for fine chemicals such as bisphenol A diglycidyl ethers. Non-oxidative microbial decarboxylation of* p*-hydroxycinnamic acids into vinylphenols involves cofactor-free and metal-independent phenolic acid decarboxylases (EC 4.1.1 carboxyl lyase family). Historically purified from bacteria (*Bacillus, Lactobacillus*, *Pseudomonas*, *Enterobacter* genera) and some yeasts (e.g. *Brettanomyces* or *Candida*), these enzymes were described for the decarboxylation of ferulic and *p*-coumaric acids into 4-vinylguaiacol and 4-vinylphenol, respectively. The catalytic mechanism comprised a first step involving *p*-hydroxycinnamic acid conversion into a semi-quinone that then decarboxylated spontaneously into the corresponding vinyl compound, in a second step. Bioconversion processes for synthesizing 4-vinylguaiacol and 4-vinylphenol by microbial decarboxylation of ferulic and *p*-coumaric acids historically attracted the most research using bacterial recombinant phenolic acid decarboxylases (especially *Bacillus* enzymes) and the processes developed to date included mono- or biphasic systems, and the use of free- or immobilized cells. More recently, filamentous fungi of the *Neolentinus lepideus* species were shown to natively produce a more versatile phenolic acid decarboxylase with high activity on sinapic acid in addition to the others p-hydroxycinnamic acids, opening the way to the production of canolol by biotechnological processes applied to rapeseed meal. Few studies have described the further microbial/enzymatic bioconversion of these vinylphenols into valuable compounds: (i) synthesis of flavours such as vanillin, 4-ethylguaiacol and 4-ethylphenol from 4-vinylguaiacol and 4-vinylphenol, (ii) laccase-mediated polymer synthesis from canolol, 4-vinylguaiacol and 4-vinylphenol.

## Introduction

**Fig. 1:**
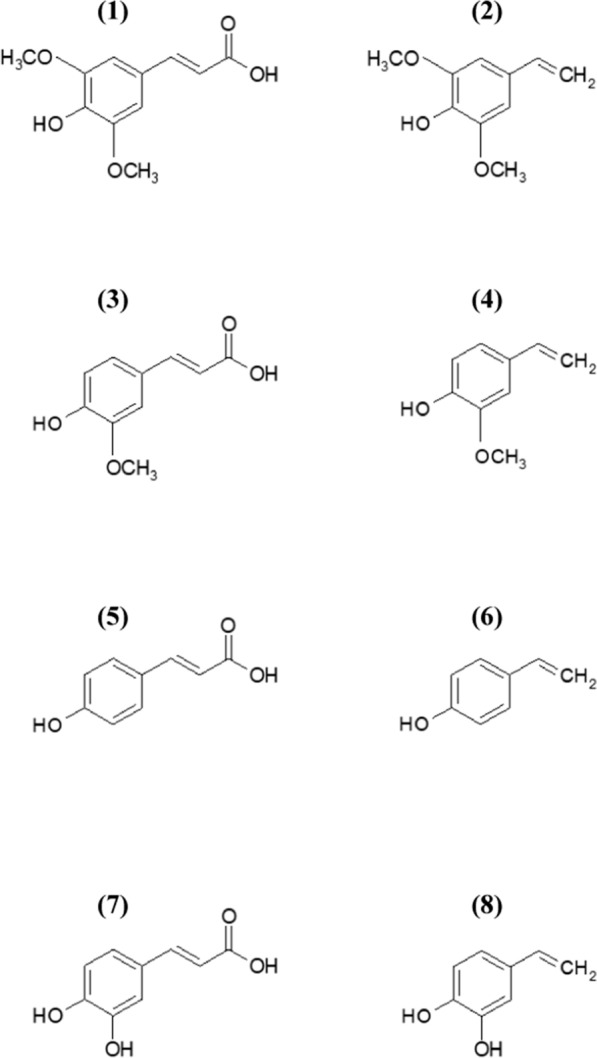
Chemical structure of *p*-hydroxycinnamic acids and their vinyl derivatives. **(1)** sinapic acid (3,5-dimethoxy-4-hydroxycinnamic acid), **(2)** canolol (2,6-dimethoxy-4-vinylphenol), **(3)** ferulic acid (3-methoxy-4-hydroxycinnamic acid), **(4)** 4-vinylguaiacol (2-methoxy-4-vinylphenol), **(5)** *p*-coumaric acid (4-hydroxycinnamic acid), **(6)** vinyl phenol (4-vinylphenol), **(7)** caffeic acid (3,4-dihydroxycinnamic acid), **(8)** 4-vinylcatechol (2-hydroxy-4-vinylphenol)

Rapeseed meal, the co-product of the de-oiling process of rapeseed/canola (*Brassica napus* L.) seeds, contains significant amounts (up to 2% dry matter) of phenolic compounds, mainly as sinapic acid esters and canolol (2,6-dimethoxy-4-vinylphenol or vinylsyringol) its thermal decarboxylation product. Other vinylphenols, including 4-vinylphenol (4-VP), 4-vinylcatechol (2-hydroxy-4-vinylphenol or 4-VC) and 4-vinylguaiacol (2-methoxy-4-vinylphenol or 4-VG) are naturally occurring aroma compounds in fermented foods and beverages, and result from microbial decarboxylation of corresponding *p-*hydroxycinnamic acids (*p-*HCAs), namely *p*-coumaric, caffeic and ferulic acids, respectively (Fig. 1). Both *p-*HCAs and their vinyl derivatives exhibit many interesting chemico-physical and biological properties which make them attractive molecules for sectors as varied as agri-food, perfumes, pharmaceuticals, fine chemicals or polymer materials, in a context where the substitution of petro-sourced chemicals for sustainable alternatives has become a major issue. From an industrial standpoint, *p*-HCAs are currently obtained from either (i) chemical or enzyme hydrolysis of agricultural biomasses followed by extraction/purification, or (ii) condensation of malonic acid with synthetic or biosourced *p*-hydroxybenzaldehydes via the Knoevenagel-Doebner reaction. However, despite the continuous improvement of synthesis routes and recovery processes, it is clear that *p*-HCAs still suffer from limited availability and high prices, which restricted their outlets to high value-added and niche markets [1]. The direct and selective synthesis of *p*-HCAs by engineered microorganisms appears today as one of most promising route for a bulk and cheap production, as shown by the recent advances in *p*-coumaric acid production from sugars [2, 3]. As for vinylphenols, their availability on the market is even more limited since they essentially result from the chemical processing, most often in harsh conditions, of *p*-HCAs (decarboxylation) or *p*-hydroxybenzaldehydes (Knoevenagel-Doebner condensation). This is particularly the case for 4-vinylcatechol and canolol whose precursors are lacking on large scale and, in a less extent, for 4-vinylguaiacol which compete with vanillin production and uses. Alternatively, the non-oxidative decarboxylation of *p*-HCAs in mild conditions using microorganisms or enzymes was also investigated over the past decade, and cofactor-free and metal-independent phenolic acid decarboxylases (PADs) were demonstrated as particularly suited and performing biocatalysts. Recently, a two-step bioconversion process combining an *Aspergillus niger* feruloyl esterase with the fungus *Neolentinus lepideus*, allowed the quantitative production of canolol from rapeseed meal [4], thus opening up new perspectives to access vinylphenols from abundant *p*-HCAs-rich biomasses such as oilseed meals, cereal brans or sugar beet and coffee pulps.

In this review, we will report the recent advances in the biotechnological production and conversion of vinylphenols, with a particular focus on canolol. In a first part, we will give a short review of the occurrence, properties, and technological significance of vinylphenols and their precursors, i.e. *p*-HCAs. Then, we will discuss the major interest of biocatalysts, and more especially microbial PADs, in the processing of hydroxycinnamic acids into vinylphenols. Finally, before concluding, we will give some examples of further transformation of vinylphenols and the still modest place of bioprocesses.

## Enzymatic conversion of hydroxycinnamic acids into vinylphenols

### Occurrence, properties, and biotechnological value of hydroxycinnamic acids

Hydroxycinnamic acids (3-phenyl-2-propenoic acids) are formed in plants via the phenylpropanoid biosynthetic pathway, which begins under the action of phenylalanine ammonia lyase (PAL) by non-oxidative deamination of l-phenylalanine into *trans*-cinnamic acid [5]. Hydroxycinnamic acids, such as ferulic acid (3-methoxy-4-hydroxycinnamic acid, FA), *p*-coumaric acid (4-hydroxycinnamic acid, pCA), sinapic acid (3,5-dimethoxy-4-hydroxycinnamic acid, SA) and caffeic acid (3,4-dihydroxycinnamic acid, CafA) (Fig. 1), are among the most abundant phenolic compounds found in plant biomass and agro-industrial by-products such as cereal brans, sugar-beet and coffee pulps and oilseed meals. FA plays a key role in plant cell-wall structure and physiology. In Gramineous plants, FA is mainly esterified in the *O*-5 position with the arabinose residues of arabinoxylans. In Dicots, it is esterified in the *O*-2 or *O*-3 position of arabinose and the *O*-6 position of galactose residues present in pectins. In contrast, the bonds between FA and lignin are essentially etherifications of the α-*O*-4 and/or β-*O*-4 type [6]. FA is found in mono- or dimeric forms. The dimeric FA forms diferulate bridges between two chains of polysaccharides [7] or between a chain of polysaccharide and lignin [8], and this structure reinforces the rigidity and integrity of the cell wall. FA is particularly abundant in common agricultural by-products such as cereal brans and sugar-beet pulp (1–4% dry matter). pCA is also particularly found in gramineous cell walls, especially in wheat straw and maize bran, where it is covalently linked to polysaccharides by ester bonds and to lignin by mainly ester but also ether bonds [6]. CafA is not bound to the various components of the plant cell wall. It is found in several soluble forms, as a dimer such as rosmarinic acid but also esterified with quinic acid to form chlorogenic acids. The family of chlorogenic acids includes: (i) monoesters of CafA, pCA and FA; (ii) di-, tri- and tetraesters of CafA, (iii) mixed CafA–FA or CafA–SA diesters; (iv) mixed esters between one or several CafA residues and an aliphatic acid such as succinic acid [9]. SA and CafA are especially abundant in rapeseed and sunflower meal, which are the main co-products of rapeseed and sunflower seed pressing and de-oiling processes. Rapeseed meals (RSM) and sunflower meals (SFM) have relatively high total phenolic compound contents (TPC), ranging from 1–2% dry matter for RSM and 2–4% dry matter for SFM. In RSM, these phenolics are almost exclusively SA esters, with sinapine as the main derivative (80% of TPC content) and the remaining 20% composed of mono-, di- and tri-sinapoyl esters of sugars and/or kaempferol [10–12]. In SFM, the phenolics are mostly CafA esters: 5-*O-*caffeoylquinic acid (chlorogenic acid) is the major phenolic compound, accounting for 70% of the TPC content, while the remaining 30% are other mono- and di-caffeoylquinic acids, plus coumaroyl- and feruloylquinic acids. SFM also contains free CafA, but it accounts for just 0.5% of the TPC content [13–15].

Hydroxycinnamic acids are bioactive molecules with many interesting properties, including antioxidant, anti-inflammatory and antimicrobial activities, among others [16–19]. Furthermore, owing to their particular structure and reactivity, hydroxycinnamic acids, as their corresponding vinylphenols obtained by decarboxylation (Fig. 1), have gained great importance in the past decade for the synthesis of new sustainable polymers and pre-polymers [20–25]. The paragraphs that follow lend special focus to SA and canolol.

The literature on SA points to pharmacological value in many areas. In vitro and in vivo studies carried out on SA have demonstrated that SA could prevent or slow progression of a wide range of diseases, from cancer and diabetes to cardiovascular disease, Alzheimer’s disease, and ischemic lesions [26, 27]. There is already two decades of research focusing on SA as a potential antioxidant, and studies have shown that it has superior effectiveness to FA, which is already in use as an antioxidant in food, beverages, and cosmetics, and equivalent effectiveness to CafA [28–30]. A number of studies have recently demonstrated that oral intake of SA can be beneficial in the prevention of many oxidative stress-related diseases. For instance, Zych et al*.* [31] studied oxidative stress associated with postmenopausal disorders and the development of cataract in estrogen-deficient rats, and showed that oral intake of SA had benefit for cataract prevention. Indeed, the intake of SA increased the content of reduced glutathione (GSH) and improved the activity of glutathione reductase and other parameters related to protein oxidation. Shahmohamady et al. [32] demonstrated that oral administration of SA in rats with Alzheimer’s disease increased antioxidant enzyme levels and decreased cell loss in the cerebral cortex and hippocampus. Furthermore, Cai et al*.* [33] used a mouse model of osteoarthritis induced by destabilization of the medial meniscus and showed that SA intake activated the signaling pathways of nuclear factor Nrf2 and heme oxygenase-1 and had a powerful anti-inflammatory effect on osteoarthritic articular cartilage. This anti-inflammatory effect was measured in vivo and in vitro as a decrease in expression of induced oxide nitrate synthase (iNOS), cyclooxygenase (COX-2), and cytokines: tumor necrosis factor TNF-α and interleukine IL-1β. SA has exhibited cytotoxic activity against various cancer cell lines, including colon cancer (HT29 and SW480 lines) and human laryngeal carcinoma (Hep-2 line) [34, 35]. Balaji then demonstrated that SA inhibited carcinogenesis by increasing the activity of enzymatic and non-enzymatic antioxidants present in the body, including catalase, superoxide dismutase (SOD) and GSH [36]. In 2018, Singh et al. showed that SA inhibited histone deacetylase, which plays a role in the initiation and progression of cancers, as well as an induction of apoptosis, thus preventing the onset of cancer [37].

### Properties and applications of vinylphenols

Otherwise, vinyl derivatives of pCA, CafA, FA and SA could also find numerous applications in food, cosmetic or pharmaceutical industries, due to their powerful antioxidant and anti-inflammatory properties [38]. An overview of the main antioxidant and biological properties of vinylphenols will be made in the following paragraphs, starting with canolol.

Analysis of antioxidant compounds in rapeseed oil by Koski et al. [39] and then Wakamatsu et al. [40] showed a correlation between the concentration of phenolic compounds and the resistance of lipids to auto-oxidation. These authors identified canolol as the phenolic compound largely responsible for the stability of rapeseed oil against oxidation. Indeed, studies by Koski et al. [39], Wakamatsu et al. [40] and Galano et al. [41] brought evidence that canolol had comparable antioxidant activity to γ-tocopherol and superior antioxidant activity to α-tocopherol, vitamin C, β-carotene, rutoside, and quercetin. Furthermore, canolol is lipophilic, which gives it better affinity with cell membranes than hydrophilic antioxidants. Studies have generally found that canolol decreases the cell apoptosis induced by oxidative stress [42–44] and acts on expression of the transcription factor Nrf-2 that regulates the expression of anti-oxidative enzymes (heme oxygenase or catalase, for example). In addition, canolol also appeared to activate extracellular signal-regulated kinases (or ‘ERK proteins’), which themselves play a role in defense against oxidative stress. Cao et al. [45] analyzed the antioxidant and anti-inflammatory effects of canolol on gastritis caused by *Helicobacter pylori*. During infection, this bacterium generates a strong immune response via the expression of cytokines (IL-1β, TNF-α), the activation of enzymes (COX-2, iNOS), and also the production of anti-*Helicobacter pylori* antibodies, and canolol was found to exert a protective role by reducing the effects of inflammation. Maeda et al. [46] used the Mongolian gerbil as an animal model of gastric carcinogenesis related to *H. pylori* infection, and found that treatment of *H. pylori*-infected animals with a diet containing canolol led to a strong reduction in gastric inflammation. Canolol was thus able to suppress the in-stomach production of 8-oxodeoxyguanosine, an in vivo marker of inflammatory pathologies resulting from the reaction of free radicals with DNA. Although canolol did not directly inhibit bacterial growth, it did have the ability to suppress inflammation and hence the cell proliferation and carcinogenesis induced by *H. pylori*. Furthermore, Jiang et al. [47] showed that canolol inhibited the growth and activated the apoptosis of human gastric adenocarcinoma cells (SGC-7901 line), thus preventing cell multiplication. Fang et al. [44] investigated the potential beneficial effects of canolol in colon cancer. Addition of canolol to the diet significantly suppressed the pathogenesis of this cancer in mice. In both cases, it seemed that the effect of canolol was associated with the inhibition of inflammatory cytokines (COX-2, iNOS, TNF). A patent, from Maeda et al. [46], protected any product (drug, cosmetic, food), formulation or method comprising canolol, intended to treat inflammation and prevent the onset of cancer. The pathologies targeted were (i): inflammatory diseases such as duodenitis, gastritis, peptic ulcer disease, bronchitis, rheumatism, hepatitis, colitis, conjunctivitis, pancreatitis, stomatitis, and pharyngitis; (ii): cancers of the stomach, colon, liver, gallbladder, bile ducts, esophagus and lungs. In the field of cosmetics, canolol could act against skin aging, burns, sunburn, and any other inflammation of the skin.

Regarding other vinylphenols, i.e. 4-VP, 4-VC and 4-VG, few works have focused on their antioxidant properties. Terpinc et al. [48] systematically evaluated their reducing power with the ferric reducing antioxidant power (FRAP) assay, as well as their ability to scavenge radicals (2,2-diphenyl-1-picrylhydrazyl radical, DPPH^•^ and superoxide anion radical, O_2_^•−^) in homogeneous solutions and in linoleic acid-based emulsion (β-carotene bleaching test). They found that 4-VC was the most efficient scavenger of stable DPPH^•^ and O_2_^•−^ radicals due to the catechol moiety, while 4-VG was the best radical chain-breaker in emulsion, highlighting thus the superior role of vinylphenol polarity over their scavenging capacity in heterogenous lipid-based systems. The corresponding hydroxycinnamic acids (i.e. *p*-coumaric, caffeic, ferulic and sinapic acids) were also evaluated in the same tests, and a similar ranking to that of the vinylphenols was obtained. Interestingly, the performance of vinylphenols in homogeneous media was found to be inferior to that of the more polar hydroxycinnamic acids, whereas the opposite situation was observed in emulsion. Fujioka and Shibamato [49] investigated the antioxidant activity of a brewed commercial coffee through the inhibition of malondialdehyde formation during cod liver oil peroxidation. The phenolic compounds present in the dichloromethane extract exhibited the strongest activity, especially 4-VG. The latter was found to be more efficient than the reference antioxidant α-tocopherol, but equivalent to synthetic butylated hydroxytoluene (BHT), with 95–100% inhibition at concentration of 0.1 g/L. In their study on the antioxidant properties of the ethyl acetate extract of commercial beers, Rahman et al. [50] reported a positive correlation between 4-VG and radical scavenging assays (p < 0.05 for 2,2′-Azino-bis-3-ethylbenzothiazoline-6-sulfonic acid ABTS^•+^ assay and p < 0.001 for FRAP assay), suggesting probable antioxidant action of 4-VG in finished beer. This strong antioxidant capacity of 4-VG was also confirmed in bulk oils [51] and in oil/water emulsion [52]. The effect of 4-VC on the stability of soybean oil-in-water emulsions stored at 40 °C for 50 days in the dark was studied by monitoring the formation of primary and secondary oxidation products [53]. Compared with α-tocopherol, 4-VC showed higher antioxidant activity at the same concentration (200 ppm). On the other hand, results regarding 4-VC + α-tocopherol mixture (100 ppm each) revealed the weak synergy of the two molecules, i.e. the low capacity of 4-VG to regenerate and recycle tocopheroxyl radical into α-tocopherol. In a similar study, Jia et al. [54] compared the efficiency of 4-VC, α-tocopherol and BHT (200 ppm each) in delaying oxidation of a stripped soybean oil heated at 60 °C in the dark. After 11 days, the oxidation extent was equivalent in sample treated with 4-VC and BHT, but significantly lower than that of the control or sample containing α-tocopherol. A powerful synergistic effect was observed with 4-VC + α-tocopherol (100 ppm each) mixture as no aldehyde was detected in the corresponding sample. These results were in agreement with those of Terpinc et al. [48] who showed that antioxidant properties of vinylphenols dramatically rely on the nature, either homogeneous (bulk oil, aqueous media) or heterogeneous (emulsion), of the system in which they were assessed.

Along with intrinsic chemical reactivity, the amphiphilic character, given on one side by the polarity of the phenolic hydroxyl and the lipophilicity of the vinyl group on the other side, likely favors interactions with interfaces in dispersed lipid systems, but also with membranes in living organisms, thus playing a role on the biological properties of 4-VG and 4-VC. For instance, Senger et al. [55, 56] linked the presence of 4-VC and some alkyl catechols to the anti-inflammatory properties of *Barleria lupulina*, an essential medicinal plant from Southeast Asia, India and southern China, and demonstrated the powerful action of these cofactors for activation of the Nrf2 (NFE2L2) cell defense pathway, both in vitro and in vivo. The first clue of possible anti-inflammatory activity of 4-VG was brought by Pongprayoon et al. [57] who extracted the molecule from *Ipomoea pes-caprae* (L.) R. Br., and then noticed a potent inhibitory effect (IC50 of 18 µM) on prostaglandin synthesis in vitro. Twenty years later, Jeong et al. [58] elucidated the anti-inflammatory mechanism in RAW264.7 murine macrophage cells stimulated with *E. coli* (055:B5) lipopolysaccharides. They found that 4-VG down-regulated the expression of some key pro-inflammatory mediators such as nitric oxide, prostaglandins (PGE_2_), iNOS and COX-2, through the suppression of (i) NF-κB and MAPK pathway activation, and (ii) histone H3 hyper-acetylation. In addition, antiproliferative effects were found on the two colorectal cancer cell lines HCT-116 and HT-29 [59], on benzo[a]pyrene-treated mouse fibroblast NIH 3T3 cells [60], as well as on human breast cancer MCF-7 cells [61] and human pancreatic cancer Panc-1 cells [62]. Finally, Rubab et al. [63] identified 4-VG as one of the main contributors of the strong antibacterial and antifungal effect of red cabbage (*Brassica oleracea var. capitata f. rubra*) chloroform extract, which was additionally proved antioxidant and non-cytotoxic (*C. elegans* chemotaxis assay). Anti-virulence properties of *Micromonospora* sp. RMA46 (a rare marine Actinobacteria from Indian mangrove) against *Vibrio cholerae* HYR14 were assessed in vitro by Sarveswari et al. [64]. The ethyl acetate extract of RMA46, containing 43 chemical compounds including 4-VG, was not bactericidal but inhibited the formation of HYR14 biofilm. Interestingly, in silico molecular docking revealed that, among all compounds, 4-VG had the highest binding capability with LuxO protein, and could therefore potentially block quorum sensing-mediated biofilm formation in *V. cholerae*.

In addition to these antioxidant, anti-inflammatory and anticarcinogenic properties, vinylphenols, and especially canolol and 4-VG, were also shown to be suitable precursors of (i) thermoplastic biopolymers and (ii) bio-based substitutes for bisphenol A diglycidyl ether [24].

Microbial decarboxylation of phenolic compounds into vinyl derivatives was first highlighted in the 1960s, cued by the presence of 4-VG and 4-VP in distillates from grain alcohol ferments such as whiskey or beer. The decarboxylation was able to occur during yeast fermentation or bacterial contamination [65]. In microorganisms, non-oxidative microbial decarboxylation of hydroxycinnamic acids notably involves intracellular enzymes called phenolic acid decarboxylases (PADs, EC 4.1.1, carboxyl lyase family), and is thought to be related to the metabolic pathways of phenolic detoxification.

### Microbial cofactor-free and metal-independent phenolic acid decarboxylases

The microbial (de)carboxylation of aromatic compounds (secondary metabolism) involves biocatalysts that are classified into three main types of enzymes: (i) divalent metal-dependent decarboxylases, (ii) prenylated flavin mononucleotide-dependent decarboxylases (UbiD superfamily), and (iii) cofactor-free metal independent decarboxylases PADs [66]. The following paragraphs focus exclusively on this third group of enzymes.

**Fig. 2:**
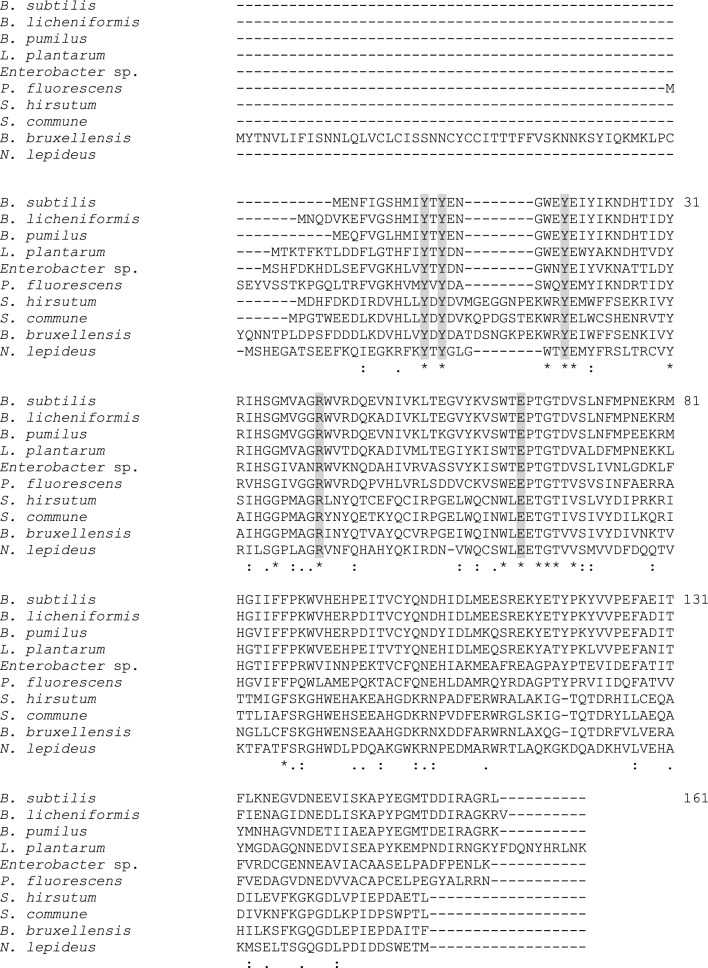
ClustalW alignment of PAD protein sequences. PADs from *Bacillus subtilis*, *Bacillus licheniformis*, *Bacillus pumilus*, *Lactobacillus plantarum*, *Enterobacter* sp., *Pseudomonas fluorescens*, *Stereum hirsutum, Schizophyllum commune, Brettanomyces bruxellensis* and *Neolentinus lepideus* (Accession Numbers in the NCBI database: PFK93232.1, ATI77534.1, WP_099727689.1, ASZ34126.1, EJF30578.1, WP_034118423, XP_007303961.1, XP_003032860.1, APP94195.1 and KZT30061.1, respectively). The sequences shared between them 16–88% similarity. The amino acids (aa) described as involved in the catalytic mechanism are indicated in grey boxed letters. These aa are highly conserved among the sequences. The numbering of aa is based on the sequence of the *B. subtilis* PAD [85]

**Fig. 3:**
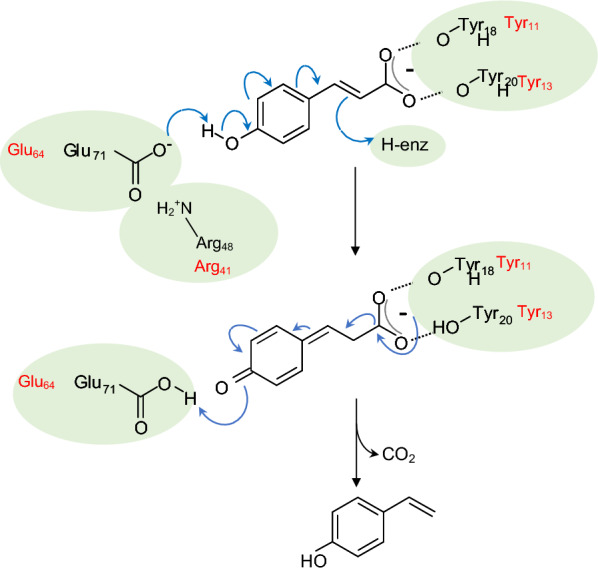
Model of the catalytic mechanism of the *L. plantarum* PAD for decarboxylation of pCA [84]. The corresponding amino acids identified in *B. subtilis* PAD are indicated in red color [85]. The carboxyl moiety of pCA is linked to residues Tyr18 and Tyr20 in this enzyme. Deprotonation of the hydroxyl group of pCA by residue Glu71 (first step) leads to the formation of a semi-quinone intermediate whose decarboxylation generates 4-vinylphenol (second step)

In 1994, a ferulic acid decarboxylase was purified and characterized for the first time from *Pseudomonas fluorescens* [67]. To date, only PADs purified from bacteria of the genera *Bacillus, Lactobacillus, Pseudomonas* and *Enterobacter* and certain yeasts (e.g. *Brettanomyces bruxellensis*, *B. anomalus*, *Candida guilliermondii*) have been thoroughly characterized in the literature [68–72] (Table 1). Bacterial and yeast PAD activity has essentially been described for the conversion of FA into 4-VG and pCA into 4-VP. A mandatory feature of the PAD substrates was that they had to possess a *para*-hydroxyl group. These PADs were homodimeric enzymes of molecular mass comprised between 40 and 46 kDa and did not require cofactors (Table 1). Their optimum temperature was between 20 and 45 °C, and their optimum pH was between 4 and 7.3. Several authors have reported inhibition/deactivation of PADs by the substrate and the product of the enzymatic reaction [68, 75]. Analysis of the structure of the crystallized PAD from the strain *Bacillus pumilus* UI-670 revealed two monomers consisting of a β-barrel structure and two α-helices [82]. The active binding site to the substrate was in a generally hydrophobic pocket, inside the barrel. The most highly-conserved residues were the hydrophobic residues that were found in this cavity of the β-barrel (Fig. 2). Both the C- and N-terminal sequences of bacterial PADs appeared to play a crucial role in the activity and substrate specificity of the enzyme [82, 83]. In bacterial PADs, the catalytic mechanism comprised a first step involving hydroxycinnamic acid conversion into a semi-quinone that then decarboxylated spontaneously into the corresponding vinyl compound in a second step [70, 84, 85]. In *Lactobacillus plantarum*, the formation of a quinone methide intermediate resulted from a deprotonation of the phenolic *p*-hydroxyl group of the substrate by Glu71, assisted by Arg48 [84]. Two Tyr residues (Tyr18 and Tyr20) were involved in the good spatial orientation of the substrate in the active site by binding the carboxylate moiety and in its subsequent decarboxylation. The same mechanism was described for the PAD from *B. subtilis* [85] (Fig. 3). In that case, the authors suggested that the protonation of the substrate C_2_–C_3_ double bond was driven by the Tyr19 residue. All these residues were found to be highly conserved in bacterial and yeast PADs [84, 85] (Fig. 2). The mobile β1–β2 loop (from Tyr11 to Ala19 in *B. subtilis*) closed over the active site in the presence of the substrate in order to restrict water ingress into the cavity [85]. It was hypothesized that the steric properties of the substituents on the aromatic ring played a more crucial role than electronic effects in explaining the specificities of PAD substrates [66].

**Table 1:** Biochemical properties of PADs described in literature

Source	Molecular mass (kDa)	Optimal pH	Optimal temperature (°C)	Substrate^a^	K_M_ (mM)	Specific activity (U/mg)	References
Bacteria							
* Bacillus pumilus* PS213	2 × 23	5.5	37	FA	1.03	72.2	Degrassi et al. [73]
				pCA	1.38	–	
* B. pumilus* DRV 52131	-	6.5	37	FA	0.31	–	Lee et al. [68]
* B. pumilus* UI-670^b^	2 × 20.4	7.3	27–30	FA pCA	7.9 –	290 –	Huang et al. [67]
* B. subtilis* I-168	2 × 22	5	40–45	FA	1.1	280	Cavin et al. [74]
				CafA	2.6	–	
				pCA	1.3	–	
* B. licheniformis* CGMCC7172	2 × 20	6	37	FA	1.55	117.82	Hu et al. [72]
				CafA	1.93	54.36	
				SA	2.45	0.46	
				pCA	1.64	157.96	
* B. amyloliquefaciens* KCTC1660	-	5.8	37	pCA FA CafA	0.7 – –	– – –	Jung et al. [75]
* B. atrophaeus* UCMB-5137	2 × 25	5.5	50	pCA FA CafA	3.45 2.57 1.68	253 178 80	Li et al. [76]
* Lactobacillus brevis* RM84	2 × 20	6	22	FA	0.78	7.7 10 10	Landete et al. [71]
				CafA	0.96		
				pCA	0.98		
* Enterobacter sp.* Px6-4	2 × 23	4	28	FA	2.4	410	Gu et al. [70]
Yeasts							
* Brettanomyces bruxellensis L2480*	2 × 21	6	40	pCA	1.22	98	Godoy et al. [69]
* Brettanomyces anomalus* NCYC615	2 × 21.8	6	40	FA pCA CafA	1.15 1.55 –	13.49 22.26 –	Edlin et al. [77]
* Candida guilliermondii* ATCC9058	2 × 20	6	25	FA pCA CafA	5.32 2.66 –	531 600 45.6	Huang et al. [78]
Fungi							
* Neolentinus lepideus* BRFM15	2 × 20	6–6.5	37–45	FA SA pCA CafA	–	–	Lomascolo et al. [79]
* Aspergillus luchuensis* ISH1	2 × 19.4	5.7	40	FA pCA CafA	8.65 6.63 39.92	–	Maeda et al. [80]
* Schizophyllum commune* DSMZ1024	2 × 21	5.5	35	FA	0.16	2.922	Detering et al. [81]

^a^FA: ferulic acid; SA: sinapic acid; CafA: caffeic acid; pCA: *p*-coumaric acid

^b^Formerly named *Pseudomonas fluorescens* by authors

–: non determined by authors

The substrate specificity of native bacterial and yeast PADs has mainly been described for FA, pCA and CafA, with studies finding a much lower specific activity for CafA in general [71, 74]. A PAD from *B. licheniformis* was shown to possess a broader substrate activity including pCA, FA, CafA, and SA decarboxylation but with the relative ratios of specific activities 100:75.6:34.4:0.3, which corresponded to V_m_ values of 268.4, 216.8, 119 and 0.78 U/mg, respectively [72]. In 2013, a wild-type *B. pumilus* PAD was engineered by directed mutagenesis, and some mutants displayed activity on SA, with the best specific activity obtained being 1.4 U/mg [86]. Curiously, the catalytic activity of this evolved enzyme towards FA decreased sevenfold at the same time. After mutations on the N- or C-terminus, an engineered PAD from *B. amyloliquefaciens* also displayed an activity on SA which, however, remained anecdotic compared to the activity on FA and pCA [87].

To date, much less literature is available regarding filamentous fungi PADs. In 2016, Xie et al. [88] described the potential of the endophytic fungus *Phomopsis liquidambari* to transform SA into canolol. In this case, canolol was further degraded via syringaldehyde, syringic acid and gallic acid through a metabolic pathway involving PAD, laccase, and gallic acid dioxygenase. With the increasing number of sequenced fungal genomes, some sequences predicted as putative PADs are likely found in annotated publicly-available fungal genomes [89], particularly in the Basidiomycota phylum, including the species *Neolentinus lepideus*, *Schizophyllum commune* and *Stereum hirsutum*. For instance, the *N. lepideus* HHB14362, *S. commune* H4-8 and *S. hirsutum* FP-91666 PAD predicted sequences showed no more than 45% similarity with those of known bacterial and yeast PADs (Fig. 2). In 2017, filamentous fungi of the *N. lepideus* species were shown to natively produce PADs with strong decarboxylation activity on both FA and SA [79]. These PADs also exhibited some, albeit weaker, activity on pCA and CafA. In particular, the production of canolol and 4-VG, in a culture medium of the *N. lepideus* BRFM15 strain supplemented daily with SA and FA, respectively, reached concentrations up to 1–1.5 g/L. The PAD from *N. lepideus* BRFM15 was also described as a homodimer, with an apparent molecular mass of about 40 kDa, and active in a pH range of 5.5–7.5 and a temperature range of 30 °C–55 °C (Table 1) [79]. In that patent, a proof-of-concept of the heterologous production of a *N. lepideus* PAD in the extracellular medium of the recombinant host *Aspergillus niger* was also successfully achieved. It is worth noting that the species *Schizophyllum commune* and *Stereum hirsutum* were capable of biotransforming only FA into 4-VG but not SA into canolol [79]. In 2018, a PAD from the filamentous fungus *Aspergillus luchuensis* was heterologously produced in *Escherichia coli* and then purified and characterized [80] (Table 1). The four conserved residue signatures of PADs were found to be Tyr 18, Tyr20, Arg48 and Glu71 in the amino acid sequence. The recombinant PAD was active on FA, pCA and CafA, with a relative activity ratio of 100:150:19, but no activity on SA was reported. A PAD from *Schizophyllum commune* was heterologously produced in the yeast *Komagataella phaffii* [81], but FA was shown to be the sole substrate of the recombinant enzyme.

### Biotechnological production of vinylphenols

Biotechnological processes for synthesizing 4-VG from FA and 4-VP from pCA have historically attracted the most research and are especially well documented for recombinant PADs from *Bacillus* species in mono- or biphasic systems. The recombinant enzymes generally remained intracellular. Consequently, the methods most intensively developed to date included the use of whole-cell biocatalysts, i.e. resting cells [76, 90–94]. However, this type of process may run into several bottlenecks, including: (i) possible cell toxicity induced by the substrate and/or product of *p*-hydroxycinnamic acid decarboxylation, (ii) the time needed for the transport system (diffusion, active transport) to enable the substrate to enter in the cells and then for the vinyl derivative to get out of the cells, and (iii) poor solubility of vinylphenols in aqueous phases. To tackle these technological challenges, biphasic aqueous-organic solvent systems have been developed in batch or continuous systems at lab or reactor scale [68, 75, 76, 92, 94, 95]. For instance, 1-octanol [75, 76], hexane [68], and cyclohexane [92] were found to be suitable organic solvents affording production yields of up to several grams of 4-VG or VP per liter. In 2013, Leisch et al. [91] tested a wide range of bioprocesses using a recombinant PAD from *B. pumilus* expressed in *E. coli*. The technologies tested included biphasic (water/toluene) whole-cell or cell-free extract biotransformations, a combination of biocatalyst with adsorbent resins for in situ 4-VG recovery, and fixed-bed bioreactors using immobilized cells. The best result enabled a productivity of 4.83 g/(L.h) 4-VG. Recently, high titers of 4-VP (17–187 g/L) from pCA, extracted from a solid lignin-rich corn stover bioprocess residue, were obtained using a recombinant PAD from *B. amyloliquefaciens* with an organic (undecanol) overlay in batch processes [94]. Continuous extraction of 4-VP in the organic phase resulted in the accumulation of 17 g/L VP with a 73% yield. Deep eutectic solvents (DES) have also emerged as alternatives to aqueous or biphasic (water/organic solvent) systems to address the poor solubility of phenolic acids in water. For instance, the *B. subtilis* PAD was tested in a reaction medium composed of choline chloride/glycerol mixed with water (1:1, w/w) [96] and produced high conversion yields (> 99%) at substrate loading of up to 300 mM. Moreover, the choice of DES influenced the acceptance of different substrates by the enzyme, as evidenced by the fact that DES favored the conversion of CafA which was only poorly decarboxylated in aqueous media. Other more original methods for producing 4-VG or VP have also emerged, including (i) an *Enterobacter* sp. PAD-based all-enzyme hydrogel for flow reactor technology [97], and (ii) in-cell crosslinked enzymes to improve *B. megaterium* whole-cell bioconversion of FA [98]. Molecular engineering enabled the creation of a *Pseudomonas putida* strain purpose-designed for producing 4-VG and 4-VP from media containing lignocellulose or lignin-derived substrates [99]. *P. putida* is originally able to release and metabolize FA from plant biomass via an operon containing *fcs* (feruloyl-CoA synthetase), *ech* (enoyl-CoA hydratase) and *vdh* (vanillin dehydrogenase) genes. By molecular engineering, the *ech* gene was deleted and further replaced by a *pad* gene to enable FA to accumulate and be converted into 4-VG.

Decarboxylation of SA into canolol could be obtained by physicochemical treatments including classical heat treatment (165 °C) of rapeseed meal (RSM), microwave treatment, or a combination of heat, pressure, and solvent extraction with alkali [100–103]. However, canolol yields remain low (0.5–0.8 mg/g RSM) and were not always reproducible, and the processes proved too random to reliably scale up.

**Fig. 4:**
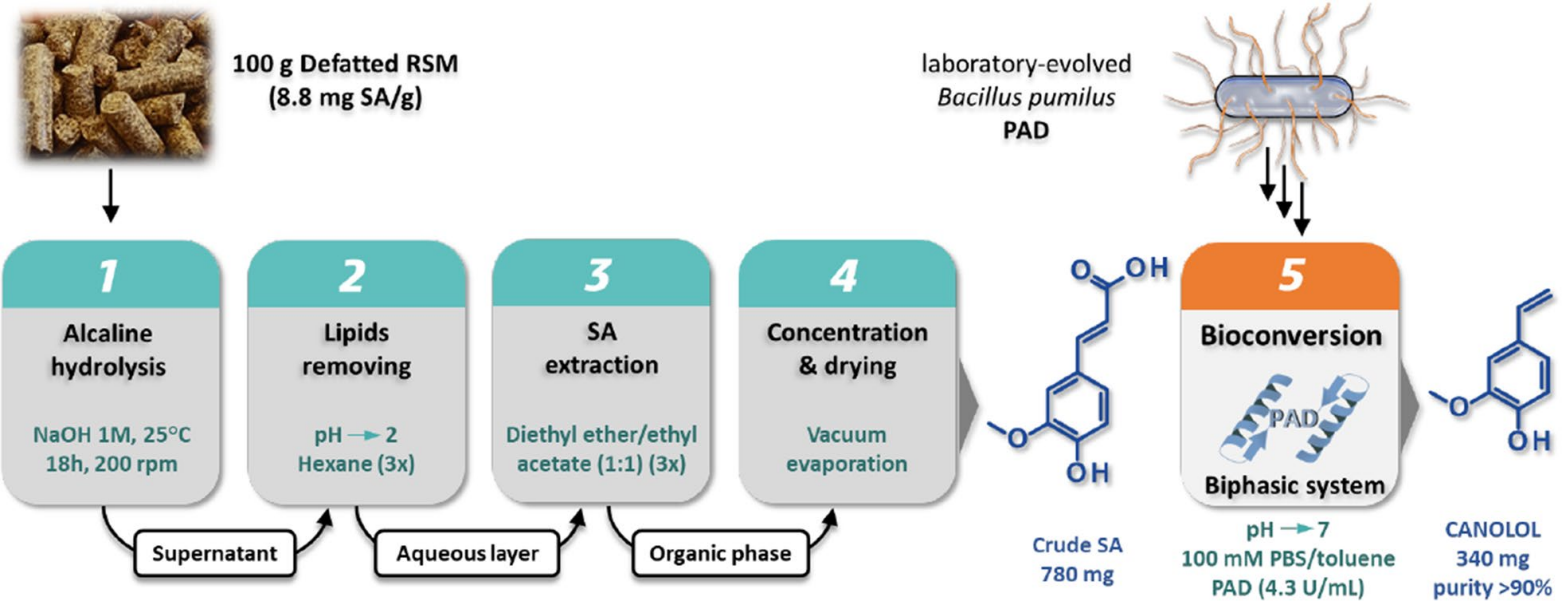
Schematic representation of the chemo-enzymatic process to produce canolol from SA from RSM. The process used an engineered *B. pumilus* PAD, according to Morley et al. [86]

**Fig. 5:**
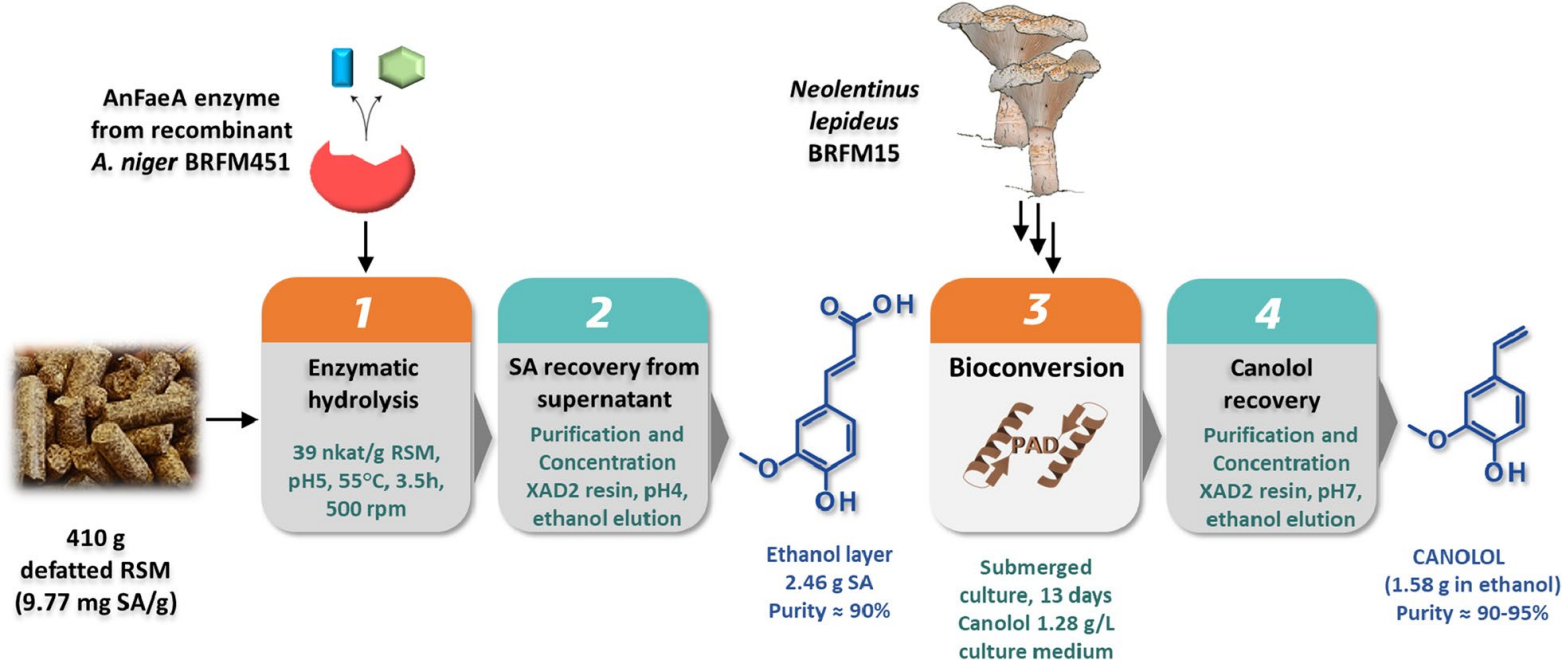
Schematic representation of the two-step biotechnological process to produce canolol from SA from RSM. The process combined the enzymatic potentialities of *Aspergillus niger* and *Neolentinus lepideus*, according to Odinot et al. [4]

In 2013, Morley et al. [86] proposed a chemoenzymatic process based on a laboratory-evolved *B. pumilus* PAD (originally inactive on SA) to synthesize canolol from SA extracted from RSM. The wild-type PAD was engineered by mutagenesis of selected amino acids including Ile85. The best mutant (Ile85Ala) showed an activity on SA of 1.4 U/mg and was chosen for the subsequent development of a two-step process including: (i) first, the chemically-driven release of free SA from RSM by alkaline hydrolysis, and (ii) second, the bioconversion of SA into canolol by the evolved PAD in a biphasic aqueous buffer/toluene system. Between these two steps, the isolation and recovery of SA from the alkaline hydrolysate was performed by solvent extractions. The different steps of the process are summarized on Fig. 4. The overall canolol production yield was 3 mg/g initial RSM [86]. A two-step bioconversion process for canolol production from RSM was later developed by harnessing and combining the complementary enzymatic potentialities of two filamentous fungi, i.e. the micromycete *Aspergillus niger* and the basidiomycete *Neolentinus lepideus* [4, 79] (Fig. 5). In the first step of the process, use of the enzyme feruloyl esterase type-A (named AnFaeA) enabled the release of free SA from the raw meal by hydrolyzing the conjugated forms of sinapic acid in the meal (mainly sinapine and glucopyranosyl sinapate). An amount of 39 nkat AnFaeA per gram of raw meal, at 55 °C and pH5, led to the recovery of 6.6–7.4 mg of free SA per gram RSM, which corresponded to a global hydrolysis yield of 68–76% and a 100% hydrolysis of sinapine. Then, the XAD2 adsorbent (a styrene and divinylbenzene copolymer resin) used at pH4 enabled efficient recovery of the released SA and its subsequent concentration after elution with ethanol. In the second step, 3-day-old submerged cultures of the strain *N. lepideus* BRFM15 were supplied with the recovered SA to serve as substrate for bioconversion into canolol via a non-oxidative decarboxylation pathway. Canolol production reached 1.28 g/L with a molar bioconversion yield of 80% and a productivity of 100 mg/(L.day). The same XAD2 resin, when used at pH7, allowed the recovery and purification of canolol from the culture broth of *N. lepideus* (Fig. 5). The overall yield of canolol production was calculated as about 3.8 mg/g initial RSM [4]. In both cases of biotechnological treatments [4, 86], a 4.1 to 6.6-fold increase in canolol synthesis per gram of RSM was achieved, compared to physical (heat) treatments.

## Bioconversion of vinylphenols into valuable compounds

**Fig. 6:**
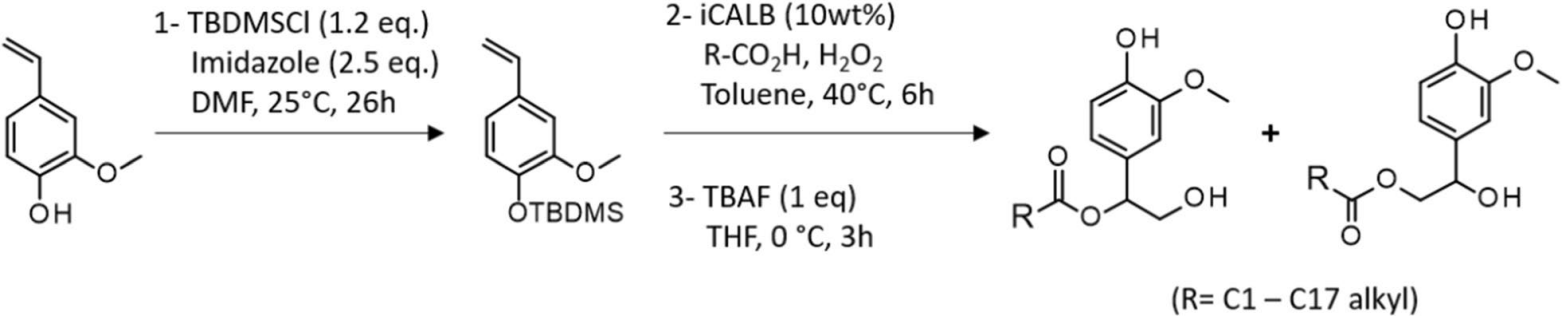
Three steps chemoenzymatic conversion of 4-VG to amphiphilic phenolic hydroxyalkyl esters, according to Zago et al. [113]

Apart from their applications in the field of flavors and fragrances, vinylphenols are considered, for more a decade, promising renewable platform molecules due to their aromatic structure and their reactivity brought by the vinyl group. However, it is worth noting that the majority of the studies published so far relies on the chemical or chemoenzymatic processing of vinylphenols from synthetic or hemi-synthetic origin. One can cites the development of surfactants [104, 105], reactive diluents [106, 107], or polymers and composites [108–112] for instance. Starting from synthetic 4-VG, Zago et al. [113], achieved the synthesis of amphiphilic antioxidants through a three steps chemoenzymatic process: (i) silylation of 4-VG, (ii) esterification of the vinylic double-bond by electrophilic addition of C2-C18 peracids generated in situ by *Candida antarctica* lipase-B and (iii) deprotection of resulting hydroxyalkyl esters (Fig. 6). Unexpectedly, silylated canolol turned out to be unreactive towards peracids. The same author also explored the production of potential sustainable alternatives to bisphenol A diglycidyl ether (DGEBA) through the conversion of 4-VG and canolol to diepoxidized diphenyls [24]: vinylphenols were first glycidylated with epichlorohydrin, and then dimerized by cross metathesis in the presence of Grubbs II catalyst. The resulting epoxy dimers were obtained in good yields with a high diastereoselectivity, and their hydrolyzed forms proved to have a relatively weak affinity towards estrogen receptor α.

Conversely, only very few studies have described the bioconversion of vinylphenols, previously obtained by the biocatalyzed decarboxylation of natural hydroxycinnamic acids. In other cases, hydroxycinnamic acids and vinyl phenols may be synthetic or natural, and the conversion of vinyl phenol chemically or enzymatically achieved (Table 2). For instance, Williamson et al. [99] performed first the decarboxylation of pCA and FA in lignocellulosic biomasses with a *Bacillus subtilis* PAD, and then the polymerization of resulting 4-VG and 4-VC through a *Trametes versicolor* laccase-mediated radical coupling. Starting from commercial CafA, Santamaria et al. [114], afforded 4-VG using a *L. plantarum* PAD, and then reduced to 4-ethylcatechol with a *L. plantarum* reductase. In the same study, 4-ethylphenol and 4-ethylguaiacol were directly obtained from 4-VG and 4-VP respectively.

**Table 2:** (Bio)processing of vinylphenols into valuable compounds

Substrate 1^a^	Catalyst	Product 1/Substrate 2	Catalyst	Product 2	References
–	–	4-VG	Engineered *E. coli*	Vanillin	Saito et al. [115]
–	–	4-VP	Engineered *E. coli*	(*S*)-4-(1-hydroxyethyl)phenol	Wuensch et al. [116]
–	–	4-VP	*L. plantarum* reductase	4-ethylphenol	Santamaria et al. [114]
–	–	4-VG	*L. plantarum* reductase	4-ethylguaiacol	Santamaria et al. [114]
CafA	*L. plantarum PAD*	4-VC	*L. plantarum* reductase	4-ethylcatechol	Santamaria et al. [114]
FA	*M. colombiense* PAD	4-VG	Pd/C	4-ethylguaiacol	Pesci et al. [117]
SA (canola)	*B. pumilus* PAD	4-VS (canolol)	AIBN or BF_3_·(Et_2_O)_2_	Poly-vinylsyringol	Morley et al. [86]
pCA + FA (corn bran)	*B. pumilus* PAD	4-VP 4-VG	BF_3_·(Et_2_O)_2_	Poly-(vinylguaiacol-co-vinylphenol)	Leisch et al. [91]
pCA, FA (plant biomasses)	*B. subtilis* PAD	4-VP 4-VG	*T. versicolor* laccase	Homo- and co-polymers	Williamson et al. [99]

^a^ FA: ferulic acid; SA: sinapic acid; CafA: caffeic acid; pCA: *p*-coumaric acid

In the two following examples, vinylphenol where biocatalytically synthesized before being chemically processed. Thus, [86, 91] investigated the cationic or radical polymerization of canolol into polyvinylsyringol, and of the mixture (4-VG + 4-VP) into poly-(vinylguaiacol-co-vinylphenol), the vinylphenols having been previously obtained from rapeseed meal and corn bran, respectively, using a *Bacillus pumilus* PAD. Interestingly, a two-step two-pot chemoenzymatic process enabling quantitative synthesis of 4-ethylguaiacol from FA was performed in a biphasic system by Pesci et al. [117]. FA was first converted into 4-VG using *Mycobacterium colombiense* PAD, and then hydrogenated to 4-ethylguaiacol using a palladium on carbon (Pd/C) catalyst. The PAD–Pd/C cascade reaction enabled 4-ethylguaiacol production at gram scale, in 70% isolated yield.

Finally, the last two examples illustrated the scarcity of papers documenting the microbial bioconversion of vinylphenols into valuable products (Table 2). The biotechnological production of vanillin from 4-VG was carried out by Saito et al. [115] using *E. coli* strains expressing Cso2 protein. Entrapping whole cells in calcium alginate beds allowed several days’ continuous vanillin production without coenzymes use. The catalyst however appeared to be sensitive towards reactive oxygen species and temperature higher than 20 °C, resulting in moderate yields. For their part, Wuensch et al. [116], unexpectedly observed the stereoselective formation of (*S*)-1-(*p*-hydroxyphenyl)ethanol derivatives, instead of the desired *p*-HCAs, when studying the carboxylation reaction of vinylphenols with different microbial PADs. Using whole cells of *E. coli* harboring *Lactobacillus plantarum* PAD in 1 M bicarbonate buffer, 4-VP was optimally converted in 82% yield with around 58% enantiomeric excess (ee). The results were however less conclusive for 4-VG (conv. 30–45%, ee 28–50%), and canolol (conv. < 1%).

## Conclusion

The microbial production of vinyl derivatives such as 4-VP and 4-VG is now well documented, and it involves bacterial/yeast PAD-mediated processes that hold evident biotechnological potential. The general near-absence of activity of these PADs towards SA has meant that the microbial synthesis of canolol has long remained an unmet challenge, despite although its high potential interest for industry. The increasing number of fungal genomes sequenced over the last decade has highlighted the presence of wild-type PADs that act on both FA and SA, thus opening up new perspectives for the fungal decarboxylation of SA into canolol. The species *Neolentinus lepideus* is a particularly promising candidate.

The PADs described in this review make attractive candidates for biotechnological applications, as they are cofactor-free and metal-independent enzymes. However, their intracellular localization remains the main bottleneck for a technological use. The proof-of-concept of the heterologous production of the *N. lepideus* PAD in the extracellular medium of the fungus *Aspergillus niger* is expected to ease this bottleneck.

It is noteworthy that the further microbial/enzymatic bioconversion of canolol into valuable compounds has so far been under-investigated and remains a fertile avenue for future exploration.

## Data Availability

Not applicable.

## Abbreviations

ABTS: 2,2′-Azino-bis-3-ethylbenzothiazoline-6-sulfonic acid
BHT: Butylated hydroxytoluene
CafA: Caffeic acid
pCA: *p*-Coumaric acid
COX-2: Cyclooxygenase 2
DES: Deep eutectic solvent
DPPH: 2,2-Diphenyl-1-picrylhydrazyl radical
FA: Ferulic acid
FRAP: Ferric reducing antioxidant power
pHCA: *p*-Hydroxycinnamic acid
IL: Interleukine
iNOS: Induced oxide nitrate synthase
PAD: Phenolic acid decarboxylase
RSM: Rapeseed meal
SA: Sinapic acid
SFM: Sunflower meal
SOD: Superoxide dismutase
TNF: Tumor necrosis factor
TPC: Total phenolic content
4-VC: 4-Vinylcatechol
4-VG: 4-Vinylguaiacol
4-VP: 4-Vinylphenol
